# The Institutional Worker

**Published:** 1918-07-20

**Authors:** 


THE
### INSTITUTIONAL WORKER
**Being a Special Supplement to "The Hospital"**

### OUR BUREAU OF INFORMATION.

**Rules for Correspondents.**

1. Every letter must be accompanied by the coupon to be cut from the back cover (inside page) of The Hospital, current issue, and must contain the name and address of the correspondent with pseudonym for publication if desired. Replies by post cannot be given save under exceptional circumstances at the Editor's discretion.

2. Letters from Approved Homes in reply to special needs published in the Bureau should state terms and full particulars, and be sent prepaid under cover to the Editor of the Bureau with name written across coupon for identification.

3. Proprietors of Homes which have not yet been entered on the List of Approved Homes, but have spare accommodation likely to suit special needs, are invited to write for an application form for registration. The fee for registration, which includes two announcements of the Home in the Bureau and other privileges, is 10s.

4. All communications to be addressed to the Editor of The Hospital, 28 Southampton Street, Strand, London, W.C. 2, and marked "Bureau of Information."

### INSTITUTION FACTS AND FIGURES.

### The Steward's Department of a Large Hospital.

#### I.—THE GENERAL DUTIES OF A STEWARD. THE PURCHASE OF FOOD.

Every large hospital has its own system of management, and what may be done in the steward's department in one hospital may come under a different department in another. It cannot, therefore, be taken that this article is meant to lay down the law as to what should be done in the steward's department, but rather to explain what is done at one of the large hospitals.

The general duties of a steward may be classified as follows: 1. The purchase and distribution of food, hardware, coal, etc. 2. Control of the porter staff. 3. Registration of patients.

##### The Steward's Committee.

In large institutions where public money is being spent the authorities have in mind that they are the trustees for the public, and consequently do their utmost to spend the money to the best advantage. In connection with so important a department as that of the steward, where there is much business done which entails the spending of large sums of money, it is desirable to have a small committee of business men, who can advise the steward as to the purchase of food, the wages to pay porters, and, in fact, deal with any difficulties in which the steward may need the co-operation of such a committee.

##### Office and Staff.

The steward's office should be situated in a central part of the main building, as business will be carried on with people outside the hospital, as well as inside. There will be the tradesmen to be dealt with, inquiries concerning patients to be answered, arrangements to be made for patients to be sent to the infirmary, and many other such things.

As the steward's duties take him to all parts of the hospital—*i.e.*, kitchen, porters' quarters, fumigator—and very often outside the hospital, the routine work of the office must be carried out by a staff of clerks. There should be a capable chief clerk, who can act for the steward in his absence, and, in addition, other clerks to deal with the clerical and routine office work. The registration of patients has to be kept up to date, the summary of the diets worked out, the time records for porters and staff to be entered, and a hundred-and-one other things to be attended to that may unexpectedly transpire during the day. The store-keeper, though not actually doing his work in the office, will have much going and coming between the stores and the office. He must be a man who can be relied upon and trusted in every way. He will have to check the goods as they are delivered, to see that the quantities ordered have been sent.

##### Method of Buying.

In a large institution the authorities generally buy either by—(1) contract; (2) the aid of professional buyers; (3) by arrangement.

1. *By Contract.*—The forms for tender are drawn up by the committee. They should state exactly what will be the terms of the contract—*e.g.* the manner of delivery of goods, the power to purchase in default, the standard of quality, rejected goods, etc. The conditions under these headings will be clearly defined. In the milk contract the committee may state that they wish the steward to visit the farms to see the kind of place their supply of milk is coming from. It will be the duty of the steward to see the contracts are carried out satisfactorily.

2. *Professional Buyers.*—These men of experience purchase at market prices, and generally on the market. The question of transport in this case has to be considered, and it is not an easy matter to get the goods delivered.

3. *By Arrangement.*—In war-time it is not left entirely to the discretion of the authorities to choose whether they shall buy by contract or in any other way. The Government invites the public to nominate firms from which they would like to secure their goods, such as meat, sugar, butter, cheese, bacon, etc.; but it is the Government who decides whether they shall deal with the firm nominated, the quantities of goods they shall order, and what price they shall pay during certain periods. It is almost impossible to compare the purchase of goods in war-time with peace-time methods, as the present conditions are quite different.

##### Diets for Patients; Food for Staff.

A hospital usually has a "dietary scale" for the patients, drawn up by the medical staff and committee. The medical officer, when putting a patient on a diet, will simply write on the "board" No. 1, 2, or 3, as the case may be; a sister will order the number of "diets," and the steward will send what each diet consists of. For instance, No. 1 diet may include fish, potatoes, milk, bread, etc., to a scale, the calorific value of each diet having carefully been considered by the committee.

The nursing staff, the resident medical staff, and the resident lay staff in a large institution have to be fed as well as the patients, and this is not a small matter when the staff is a large one. It should be left to the discretion of the steward, with the help of his chef, to provide as wholesome and varied a menu in as economical a way as is consistent with the work the staff are doing. Members of the steward's committee should visit the meals from time to time, in order to see that there is plenty of good, wholesome food provided, and that it is being served efficiently.

The steward will order the meat, fish, bread, milk, and perishable goods daily according to his requirements.

##### Delivery and Inspection.

Meat, fish, and vegetables should be delivered direct to the kitchen; milk, bread, groceries, and hardware should be delivered to the stores. In addition to the daily orders there will be arrivals of odd things, such as crockery and articles not kept in

(*Continued on page 2, col. 3.*)

### JULY QUESTIONS.

### I. War Vacancies.

**Give model "Terms of Appointment" applicable for the substitute of a hospital's administrative official who has been called to the Colours. Provision to be made for the possible return to duty of the official previous to the termination of the war and the filling of the appointment in case of the non-return of the official concerned. Describe fully any experiences in this connection that may prove of value to hospital managers generally.**

### 2. Bedroom Suppers for Nurses Off Duty.

**In those hospitals having no kitchen in the Nurses' Home what arrangements can be made whereby the sisters and nurses who are off duty in the evening may have the comfort of supper in their bedrooms? What method may be adopted in order to avoid the dining-room maid being faced by a more or less depleted pantry when she wishes to lay breakfast? (The difficulty seems to be not so much as regards the food as the crockery.)**

#### RULES.

The following rules must be observed:—

1. Contributions must be written on one side of the paper. Brevity and terseness are desirable features. The MSS. must bear the name and address of the sender and be accompanied by coupon to be cut from the back cover (inside page) of the current issue of The Hospital. A pseudonym must be chosen if the name is not to be published.
2. Contributions must be addressed to the Editor of The Hospital, Institutional Worker Supplement, 28 & 29 Southampton Street, Strand, London, W.C. 2, should reach him before the end of the current month, and be marked in the left-hand corner "Facts and Figures."

**A minimum payment of Five Shillings will be made for each published answer.**

### QUESTIONS INVITED.

**In connection with our Question Box, we offer every month five shillings each for the two best questions which are sent in for consideration. The questions may be on any institutional subject and concern any department, from the secretary's to the porter's, or the matron's to the domestic staff; the one essential is that they must be practical and deal with points that have a definite relation to hospital or institutional administration, upkeep, finance, or management. Points relating to artificers' work, housekeeping, the laundry, out-patients, and other practical matters will be welcome. It is hoped that thus, when difficult or doubtful points arise in the course of their work, institutional workers will be encouraged to put them in the form of a question and send them to the Editor, The Hospital Bureau of Information, 28 & 29 Southampton Street, Strand, W.C. 2, marked "Question Box."**

**The best questions will be published in due course and our readers will be given the opportunity of answering them. Thus all institutional workers may be able to co-operate with the view of helping the work and smoothing the difficulties of each other. In short, we want the Question Box of the *Institutional Worker* to be freely used by every worker habitually.**

### ENQUIRIES AND ANSWERS.

### *THE HOUSEKEEPERS' DEPARTMENT.*

#### Dried Milk and Cream.

You need have no hesitation in purchasing the dried milk prepared and sold by the West Surrey Central Dairy Company, Limited, Guildford. The food value of the milk is in no way destroyed in the process of evaporation. It is a reliable food and infinitely better to administer to children than milk of a doubtful purity, and in cases where the parents cannot be relied upon efficiently to pasteurise the milk. Dried cream is also prepared by the same firm, and is eminently reliable. —Matron.

### *MISCELLANEOUS.*

#### Clinical Thermometers.

It is extremely difficult to make any recommendation as regards the purchase of thermometers. Our experience is that even the most expensive cannot always be relied upon; we advise you to give a reasonable price to a good firm of instrument-makers willing to undertake the exchange of defective instruments.—Secretary.

#### Chiropodists' Dressings.

Corn and bunion shields and the various plasters, paddings, and dressings used by chiropodists may be obtained of Leslie's, Limited, 18 Eldon Street, London, E.C. 2.—Chiropodist.

#### A Report on Dried Milk for Infants.

You can obtain a Résumé of a Report on Dried Milk for Infants to the Local Government Board from H.M. Stationery Office, Imperial House, Kingsway, W.C., 2. The price is 1½d. —Creche Matron.

#### An Excellent Jar-cover.

In many hospital dispensaries the lid of the ointment jar is frequently conspicuous by its absence. The reason for this is that the lid is not attached to the jar. We suggest that a trial be made of an excellent little device which may be obtained of J. Davies and Company, of 43 Basinghall Street, London, E.C., known as "Lid-on." This is an adjustable spring lid which can be clipped to the side of the jar. Although perhaps not so serviceable for an ointment jar as a lid with a flange, it is better than no lid at all. For jugs it is excellent.—Luna.

#### Council on Health and Housing.

You probably refer to the Mansion House Council on Health and Housing, which is amalgamated with the National League for Physical Education and Improvement. The address is 4 Tavistock Square, W.C. The object of the Council is to improve the sanitary condition of the homes of the poor and to promote the formation of and assist health societies for checking the spread of tuberculosis, infantile mortality, and to promote general personal hygiene.—Interested.

### *WAR MATTERS.*

#### Lady Clerical Workers Required.

An appeal is being made for lady clerical workers by the Central Prisoners of War Committee, 4 Thurloe Place, S.W. 7. The work involved in indexing the names of 100,000 prisoners is considered as important as packing the parcels, as it is most necessary that the addressing is correct. A small salary will be given where it is required.

#### Volunteer Field Ambulance Sections.

The War Office has called upon the County of London to provide a number of Volunteer Field Ambulance Sections for service at home in the event of an emergency rising, and it is required to raise the *personnel* for these sections from the members of the Men's Voluntary Aid Detachments in the county. We suggest that you write to the County Director, 24 Eaton Square, S.W. 1, who will give you all necessary information upon the subject.—V.A.D.

#### Nurses for Home Hospitals.

Many more nurses are required for service in the Home hospitals, and nurses with three or even two years' training are eligible and should apply to the Matron-in-Chief, 83 Pall Mall, S.W. 1.—Constance.

### *THE SICK AND IN NEED.*

#### Case of Neglect of Duty.

Your best course would be to write, giving full details of the case, to the Central Midwives Board, Caxton House, Tothill Street, Westminster, S.W., who will, no doubt, inquire into the case you refer to.—Visitor.

### THE STEWARD'S DEPARTMENT OF A LARGE HOSPITAL.

(*Continued from page* 1.)

stock, so that all the time, more or less, there will be deliveries of goods to check.

When the meat, fish, and milk arrive it is an important duty of the steward to see that they are good and fit for consumption. If there is any doubt about them being so, they must be rejected. The milk from all the churns at each delivery should be tasted before being mixed and sent to the wards. Periodically samples of the milk should be sent to be analysed by the analyst appointed by the committee. At the same time it is only fair that samples of the same milk should be sent to the company, in order that they may have it analysed too. The steward should, as far as possible, test the milk, and see the meat and fish on arrival himself. This, however, is not always possible, as he may be engaged elsewhere at the time. It only proves how necessary and important it is that the storekeeper and chef should be competent men to act and judge in the steward's absence.

(*To be continued.*)

